# Mixing instabilities during shearing of metals

**DOI:** 10.1038/s41467-017-01879-5

**Published:** 2017-11-20

**Authors:** Mohsen Pouryazdan, Boris J. P. Kaus, Alexander Rack, Alexey Ershov, Horst Hahn

**Affiliations:** 1 Institute of Nanotechnology, Karlsruhe Institute of Technology, 76021 Karlsruhe, Germany; 2 Institute of Geosciences and Center for Computational Sciences, University of Mainz, 55099 Mainz, Germany; 3 European Synchrotron Radiation Facility, 38043 Grenoble, France; 4 Institute for Photon Science and Synchrotron Radiation, Karlsruhe Institute of Technology, 76021 Karlsruhe, Germany; 5 Laboratory for Applications of Synchrotron Radiation, Karlsruhe Institute of Technology, 76131 Karlsruhe, Germany; 6 Joint Research Laboratory Nanomaterials at Technische Universität Darmstadt, 64287 Darmstadt, Germany

## Abstract

Severe plastic deformation of solids is relevant to many materials processing techniques as well as tribological events such as wear. It results in microstructural refinement, redistribution of phases, and ultimately even mixing. However, mostly due to inability to experimentally capture the dynamics of deformation, the underlying physical mechanisms remain elusive. Here, we introduce a strategy that reveals details of morphological evolution upon shearing up to ultrahigh strains. Our experiments on metallic multilayers find that mechanically stronger layers either fold in a quasi-regular manner and subsequently evolve into periodic vortices, or delaminate into finer layers before mixing takes place. Numerical simulations performed by treating the phases as nonlinear viscous fluids reproduce the experimental findings and reveal the origin for emergence of a wealth of morphologies in deforming solids. They show that the same instability that causes kilometer-thick rock layers to fold on geological timescales is acting here at micrometer level.

## Abstract (web summary)

The mechanisms behind deformation of multiphase solids are elusive. Here, the authors use X-rays and simulations to show that the same mechanisms causing rocks to fold occur at the micrometer scale in dual-metal lamellas of Ag/Cu and Al/Cu under high-pressure torsion, leading to vortices formation.

## Introduction

Most metals we deal with in everyday life are alloys consisting of different phases. As these alloys experience shear deformation, either during fabrication or under service conditions (such as frictional wear), their phases undergo microstructural refinements. High shear strains can cause extreme morphological development of the phases^1^, to such an extent that can eventually lead to complete mixing and homogenization^2–4^.

By its very nature, shear-induced deformation and ultimate mixing entails dynamic mass redistribution across length scales that range from the atomic to continuum dimensions. Computer simulations of the process have so far mostly addressed the atomic scale^5–9^. Due to computational limitations, however, such simulations can normally simulate only a few nanoseconds of deformation with a relatively small number of atoms, and thus disregard processes taking place on larger scales. Moreover, the experimental verification of mixing models remains a serious challenge, since the results can typically only be imaged at the end of an experiment^10–12^, which does not reveal the complete temporal evolution of the process. As a result, we currently have an incomplete understanding of the deformation and mixing mechanics of multiphase systems, and lack a unified model that captures the actual length and timescales involved in the process.

Here, we first introduce a strategy that reveals the morphological development of deforming multiphase solids in a continuous manner at micrometer scale. For this purpose, high-pressure torsion (HPT) is applied on Ag/Cu and Al/Cu multilayers and the materials are subsequently imaged using three-dimensional (3D) X-ray synchrotron tomography. The results disclose a host of morphological events including formation of folds and vortices within deforming solids. In the next step, a numerical model is proposed and the experimental findings serve as a reference to evaluate it. The simulations show that the seemingly complex experimental observations can be reproduced using only a few material parameters such as viscosity and stress exponent as input, and identify the underlying mechanism to be scale-independent. The model reveals that the shearing instability in our experiments is similar to geological systems, which takes place across large length scales and over millions of years. While the experiments here are performed on multilayers under shear, the model is not limited to such arrangement and can be applied to any material system regardless of its morphology. This makes the model a versatile tool for studying a broad range of materials and material processing techniques.

## Results

### Capturing the morphological evolution

Besides the in situ experiments, which are not technically feasible for ultrahigh strains, capturing the evolution in detail entails the following steps: using innumerable identical initial samples, performing shearing experiments on each for slightly different levels of strain, and subsequently imaging all the samples, which is a tedious and impractical task. Our experimental method is a variation of this approach by performing all different levels of straining on a single sample and in a single experiment, followed by imaging.

**Fig. 1:**
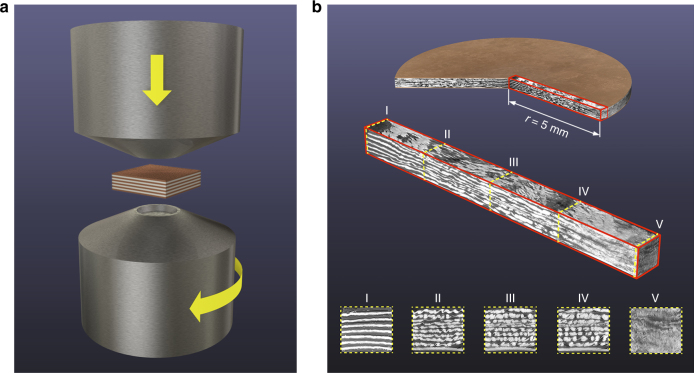
Schematics of the deformation method, the multilayer, and the volume section used for tomography examination. **a** A schematic drawing showing the high-pressure torsion (HPT) setup and a multilayer sample placed between the anvils of the apparatus. The process applies a shear stress parallel to the layers and results in the formation of a disk-shaped sample. **b** A parallelepiped-shaped volume section, cut out of the processed disk and subsequently imaged by 3D X-ray synchrotron tomography. The tomographic images show selected cross-sections of the volume element, cut at different distances from the center of the disk, with (I) representing the center (and thus having experienced the lowest strain) and (V) typifying the microstructure at the edge of the disk (and thus having been subjected to the highest strain)

Our strategy involves combining HPT experiments^13^, with three-dimensional X-ray synchrotron tomography. Two model material systems were used for this purpose: silver/copper (Ag/Cu), which is an example of a nearly immiscible system and aluminum/copper (Al/Cu), which is partially miscible. In both cases a 525-µm-thick multilayer stack, representing a multiphase alloy, was created from individual 25-µm-thick foils of each material (Methods). Next, the multilayer stack was shear deformed using the HPT method (Fig. 1a), during which two anvils are pressed against each other (4.5 GPa) with the sample in between while one anvil is rotating (1 r.p.m.). This results in the formation of a disk-like sample. The total amount of shear strain, *γ*, applied to the disk varies with its radius *r* according to

1 $${\it{\gamma }} = \frac{{2{\it{\pi }}rN}}{t},$$

where *N* is the number of revolutions and *t* is the thickness of the disk (400 µm)^14^. The amount of shear strain increases linearly from the center (*r* = 0 mm) to the circumference (*r* = 5 mm). Prior to shearing, all cross-sections in the stack are identical. During the HPT, due to existence of a strain gradient along the radius, each radial cross-section (i.e., plane with radius as its normal) is sheared to slightly different extent in proportion to its *r*-value. This causes a gradual evolution of morphology in the stack from its original phase-separated state to a relatively mixed-phase state, in the resulting sample disk (Supplementary Fig. 1). The interfacial and morphological evolution undergone by the stack can then be backtracked by looking at successive cross-sections at different radii (*r*), corresponding to different levels of strain, using 3D X-ray synchrotron tomography (Methods) (Fig. 1b). Several representative cross-sections show the structural changes that occurred with increasing strain, from the un-deformed center to the relatively mixed state (at micrometer scale) at the edge of the deformed disk. The structural changes occurring across the vicinal cross-sections in our experiments are so slight that the structural evolution due to shearing can be visualized in the form of a movie by using the same principle as flipbooks: a series of slightly shifted slides played in sequence.

**Fig. 2:**
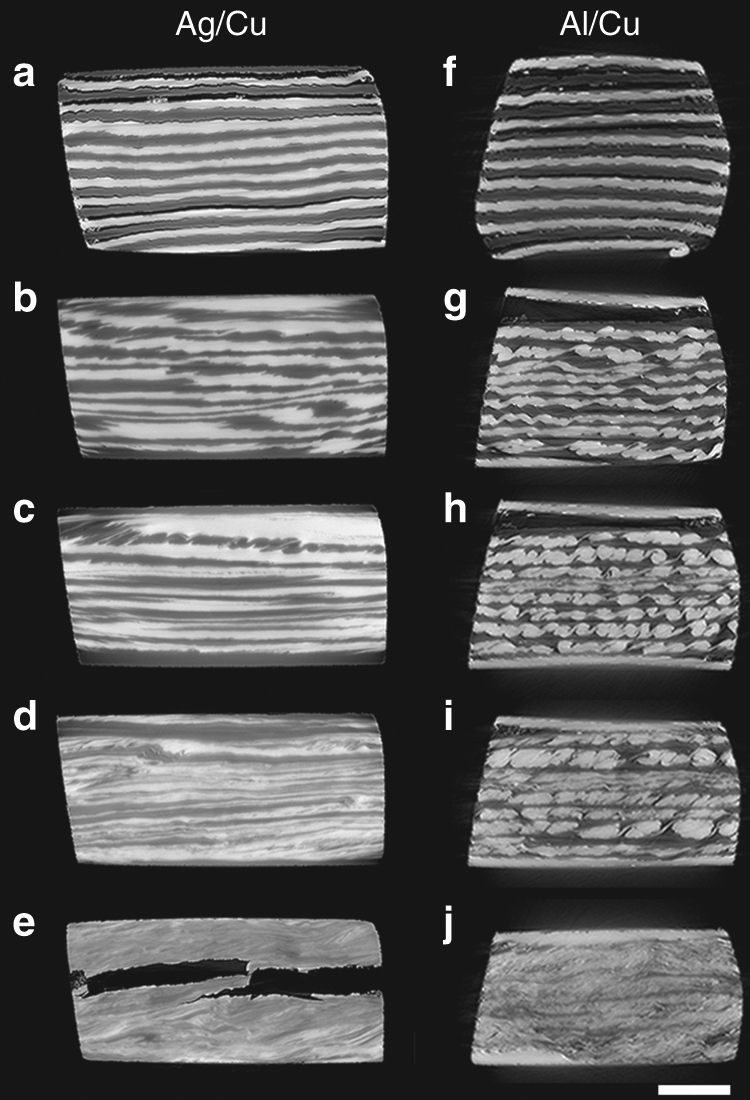
Morphological evolution of multilayers upon shearing acquired by 3D X-ray synchrotron tomography. **a**–**e** Selected snapshots of Ag/Cu-multilayer sample that had been shear-deformed parallel to the layers at 1 r.p.m. for five HPT revolutions, corresponding to a maximum strain of 393. The brighter phases are Ag. Here, the Cu layers predominantly delaminate before mixing at micrometer scale takes place. See Supplementary Movie 1 for the entire deformation sequence. **f**–**j** Selected snapshots of the Al/Cu-multilayer sample, shear-deformed parallel to the layers at 1 r.p.m. for three HPT revolutions, corresponding to a maximum strain of 236. The brighter phases are Cu. Here, the Cu layers initially fold in a quasi-regular manner and subsequently evolve into periodic vortices, before mixing at micrometer scale takes place. See Supplementary Movie 2 for the whole sequence. Note that in both sequences, the overall strain increases when going from the top to the bottom of each panel. The scale bar is 200 µm. The dark area within **e** is a crack and it occurred after HPT deformation, during the cutting of the disk for tomography examination

A representative experiment of an Ag/Cu multilayer system deformed for five revolutions to an equivalent maximum strain of 393 at the outer rim (Fig. 2a–e, and Supplementary Movie 1) reveals that delamination, i.e., the splitting of individual layers during shearing of the disk sample is the predominant behavior. Initially, the interfaces are flat (Fig. 2a), but with increasing strain they become rougher (Fig. 2b), and start to delaminate. As strain proceeds, the formation of successively finer new layers is observed (Fig. 2c–e). Up to intermediate levels of strain, the layers are predominantly parallel to each other and preferentially oriented along the shear direction. At high levels of strain, however, the arrangements of the layers take on a convoluted morphology.

The same experiment on Al/Cu multilayer system reveals a distinctly different behavior (Fig. 2f–j and Supplementary Movie 2). Initially, the Cu layers develop a folded or wavy-type structure (Fig. 2g) that eventually gives rise to a series of clockwise-rotating vortices (Fig. 2h). Neighboring vortices are connected to each other through narrow tilted tails. With increasing strain, the adjacent vortices appear to merge, which eventually result in the formation of larger vortices (Fig. 2i). In a few layers within the stack, however, a local evolution is observed that corresponds to a higher degree of deformation (Fig. 2h, i). At even higher strains, the morphology becomes tangled and turbulent-like, i.e., comparatively mixed (Fig. 2j), similar to the Ag/Cu system (Fig. 2e). Thus, the final states in mixing of the layers are similar for the two materials systems, but the pathways leading to these morphologies are distinctly different (see also Supplementary Fig. 2 for a more detailed analysis of the evolution for three successive Cu layers, within the Al/Cu multilayer sample).

Interestingly, many structural features observed during shear deformation of Al/Cu multilayer have some resemblances to the vortices emerging from Kelvin–Helmholtz (KH) instabilities in fluids (Supplementary Fig. 3), which have been ascribed by some researchers to be the underlying physical mechanism in formation of fold patterns on metals surfaces or interfaces during sliding^5, 15–17^. However, KH instabilities develop as a result of inertial forces in rapidly flowing, low viscous fluids that have high Reynolds numbers. Deforming metals, on the other hand, have much larger viscosities and as a result a very small Reynolds number, which implies that the KH instability is not a viable mechanism here (Supplementary Note 1). In addition, Beckmann et al.^18^ have recently associated such behavior in metals to dislocation-mediated plastic flow in grains with suitably oriented slip systems, which is distinctly different from KH instabilities in fluids. Nevertheless, since our observations here extend to length scales much larger than the grain sizes, there should be another overall mechanism in action. Furthermore, none of the aforementioned explanations justifies the delamination behavior in the Ag/Cu system, which has also been observed at the nanometer scale in sheared equi-axed grains^19, 20^, implying that it is not necessarily restricted to multilayer structures or large length scales.

### Numerical simulations

A similar folding and shearing behavior as in our experiments well known to occur in geological systems, where rocks behave as (power-law) viscous fluids on a million-year timescale and are often deformed in kilometer-scale folds during mountain-building processes. Mechanically, these systems are well-understood and can be shown to be caused by layer-parallel compression of a highly viscous layer embedded in a lower viscous matrix^21^. In multilayer systems, instabilities are known to grow faster^22, 23^.

**Fig. 3:**
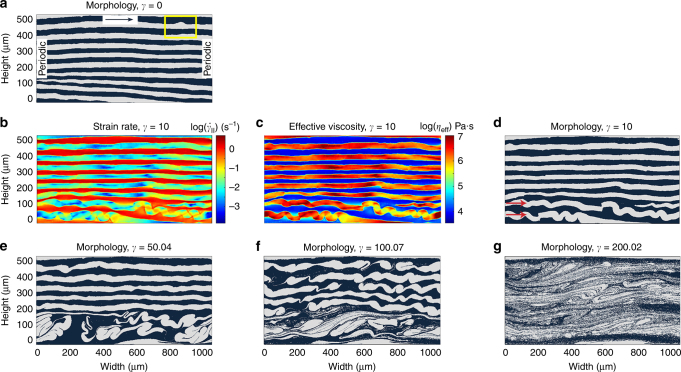
Finite element simulation of shear deformation in a multilayer system. **a** The computational cell showing the initial arrangement of the layers and the direction of shear at the top. Black (lower characteristic viscosity) and white (higher characteristic viscosity) layers denote the two different phases in each experiment, and the model domain is comparable in size to that of the samples used in 3D X-ray synchrotron tomography experiments. The evolution of the yellow marked area is shown in Fig. 4a. **b**–**g** Simulation results for (VC = 10, *n* = 3). **b**–**d** Local strain rate (${\dot{\it{\gamma }}}_{{\mathrm{II}}}$), effective viscosity (*η*_eff_), and morphology maps at shear strain *γ* = 10, respectively. The arrows in **d** point to two folding layers. **e**–**g** Evolution of morphology with the increase of shear strain (*γ*) as indicated. See Supplementary Movies 3–5 for the dynamical evolution of morphology, strain rate, and effective viscosity maps, respectively

In order to understand the mixing instabilities during shearing of metallic multilayers, we therefore performed finite element simulations of the experimental setup using a continuum mechanics approach (see Methods). Since the deformation rates are low, inertial forces are negligible and the metals behave as slowly deforming incompressible nonlinear viscous fluids, which we assume to follow the power-law relationship:

2 $${\it{\eta }}_{{\mathrm{eff}}} = {\it{\eta }}_{\mathrm{0}}\left( {\frac{{{{\dot \gamma }}_{{\mathrm{II}}}}}{{{{\dot \gamma }}_0}}} \right)^{\frac{1}{n} - 1},$$

where *η*_eff_ is the effective viscosity (Pa·s), *η*_0_ the reference viscosity (Pa·s), ${{\dot \gamma }}_{{\mathrm{II}}}$ is the second invariant of the strain rate tensor, ${{\dot \gamma }}_{\mathrm{0}}$ a reference strain rate (taken as 1 s^−1^ here) and *n* a stress exponent. Experimentally determined values of viscosity of metals vary over several orders of magnitude^24^, and hence we varied it in our simulations. Here, a characteristic viscosity of *η*_0_ = 10^5^ Pa·s (see Methods) is assigned to the black phase (Fig. 3a), representing Ag or Al, whereas the white phase has a viscosity that is larger by a factor VC (viscosity contrast). The power-law exponent *n* is varied between 1 and 5, consistent with the typical range for pure metals and solid solutions^25^. We use a microscopic image of a cross-section from a real sample as initial condition for the simulations, and apply a simple shear boundary condition with a strain rate of 1.3 s^−1^, which corresponds to the maximum experimental strain rate (see Methods) (Fig. 3a).

The results of a typical numerical simulation for VC = 10 and *n* = 3 show many similarities with the experimental results (Fig. 3b–g, and Supplementary Movies 3–5). Initially, at strains as low as 10 (Fig. 3b–d), the highly viscous layers in the region of the model with higher compressions (below ~200 µm in Fig. 3a) deform and fold. After an initial phase of folding (Fig. 3d, e), shearing occurs and other layers are affected (Fig. 3f), until the whole assemblage is mixed (Fig. 3g).

**Fig. 4:**
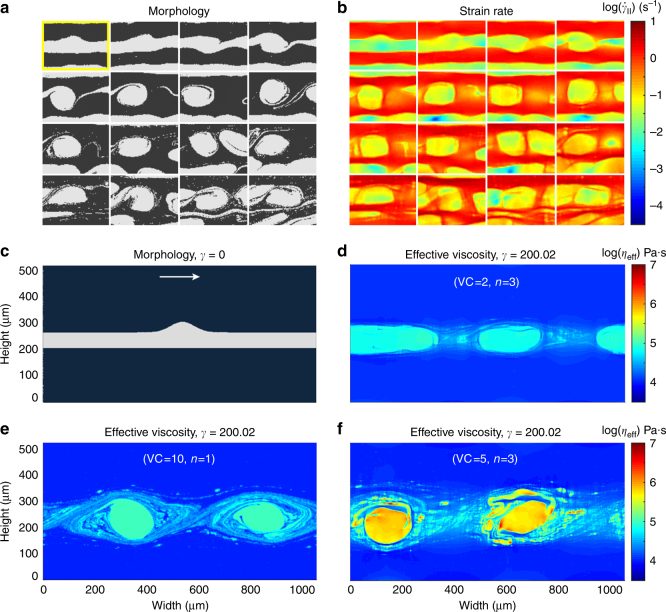
Evolution of a perturbation with shear. **a**,** b** The perturbation in the initial multilayer cell marked in the yellow box (Fig. 3a), but for a simulation with (VC = 5, *n* = 3). The shear increases from top-left to bottom-right. **a** Morphology map. **b** Strain rate map. The selected snapshots are obtained by tracking the feature in the simulation cell using an image correlation technique. See Supplementary Movie 6 for the dynamical evolutions of morphology and strain rate. **c**–**f** Simulation of a single layer with a symmetrical perturbation. **c** The computational cell showing the initial layer with higher characteristic viscosity (white) embedded within a matrix with lower characteristic viscosity (black). The arrow shows the direction of shear. **d**–**f** Effective viscosity (*η*_eff_) maps at shear strain *γ* = 200.02 for three cases as indicated. Note that the overall effective viscosity contrast between the two phases increases from **d**–**f**

The temporal evolution from a layer to a vortex structure can be illustrated by following a single location through the computational domain, which shows that layer folding occurs before a vortex is formed (Fig. 4a, Supplementary Movie 6). Mass conservation law necessitates an accelerated horizontal flow above the crests and below the troughs of the protruded layer, which is confirmed by tracking the local strain rate evolution with ongoing deformation (Fig. 4b and Supplementary Movie 6). The development of vorticity induces vertical velocity perturbations that amplify the original protrusion, resulting in positive feedback and growth of the perturbation and ultimately formation of a vortex.

The experimental observations clearly reveal that delamination and formation of vortices are the dominant features displayed by the Ag/Cu and Al/Cu multilayer systems, respectively. These entirely different features might lead to the conjecture that the structural evolution in those systems has different origins. However, a series of systematic numerical studies illustrate that delamination and high-wavelength folding are in fact two manifestations out of a range of possible morphologies that can emerge (Supplementary Figs. 4 and 5). For a given constant value of *n*, in systems with low VC values (and small effective viscosity ratios), delamination is dominant while very high VC values lead to fold formation with large wavelength values (Supplementary Figs. 4, 6, 7, and Supplementary Note 2). In systems with intermediate VC values, different degrees of delamination and rolling (vortex-like structures) are observed. A similar trend was also observed by varying *n* at constant VC value, where delamination was predominant at low *n-*values; high-wavelength folds derived at high *n*-values, whereas different degrees of delamination and rolling were seen at intermediate values of *n* (Supplementary Fig. 5).

In order to better understand the underlying physics and dismiss the effect of the neighboring layers, we performed a series of simulations using a single layer with a symmetrical perturbation having a higher characteristic viscosity embedded within a matrix of lower viscosity (Fig. 4c). The results are shown in Fig. 4d–f, which correspond to effective viscosity maps after a strain of 200 for three cases (VC = 2, *n* = 3), (VC = 10, *n* = 1), and (VC = 5, *n* = 3), respectively.

The first remarkable result is that in all cases vortices are formed, despite of the fact that in multilayer arrangements, the same parameters as in Fig. 4d, e led to predominant delamination of the system (Supplementary Figs. 4 and 5). The formation of vortices is independent of the rather ideal initial morphology here, since the same simulations on a typical single layer with rough interfaces, as in Fig. 3a, lead to similar behavior but with higher number of vortices (Supplementary Fig. 8). This discrepancy between the behavior of a single layer and a stack of layers can be explained by noting that in Fig. 4d–f, the thickness of the affected area in each case is different, which implies that if identical layers were stacked, neighboring layers would affect the evolution of each other to different extents, depending on the (VC,*n*)-combination of the system. In case of Al/Cu multilayers (Fig. 2), the adjacent layers mostly do not influence each other until the final stages of deformation and, as a result, vortices continue to develop. In Ag/Cu multilayer, the conditions are different and neighboring layers do affect each other. Hence, delamination observed in the Ag/Cu system is not intrinsic but rather caused by the stacking of thin layers. This means that folds and vortices can develop where the mechanically strong layer is embedded in thicker layers of weaker material. An example of this can be seen toward the top of Fig. 2c, where the Cu layer is folded when it is surrounded by thicker Ag layers.

Another noteworthy feature is that, as the overall effective viscosity contrasts between the phases increases from Fig. 4d–f, the tendency of the highly viscous phase to stay coherent increases (see also Supplementary Note 2).

## Discussion

The morphological development patterns, predicted by the simulations, are intriguingly similar to those captured experimentally. The model identifies the viscosity contrast and the stress exponent of the phases, as well as the strain rate as the key factors driving the microstructural evolution. Furthermore, the simulation results have demonstrated how the entire morphological evolution of any material system can be affected by the initial morphology, interfacial irregularities, and the relative orientation of the phases with respect to the shear direction. Since our numerical simulations do not include scale-dependent terms, effects such as the critical diffusion distance or critical slip distance for frictional memory appear to be of secondary importance for forming the structures we observe in our experiments.

Comparing previous atomic scale simulations with experiments (which naturally cover the whole range of events)^2^, indicates that indeed large-scale phenomena contribute more to the mixing process than merely atomic scale processes. In other words, by disregarding large-scale events in the atomistic simulations, the system requires substantially larger amounts of strain for complete mixing than what is experimentally observed. We also conclude that in the continuum regime, mechanical instability due to shearing is the overall mechanism leading the microstructural evolution, and the atomistic deformation processes, involving various dislocation-based activities or deformation twinning^26, 27^ in crystalline systems, are in fact different means for the system to accommodate the imposed instability. A similar behavior is expected in amorphous systems such as bulk metallic glasses; in fact the nature of both the experiment and the modeling method performed here make them suitable for studying this class of less-understood materials, as well.

The combination of high-pressure torsion, 3D X-ray synchrotron tomography imaging, and numerical modeling thus allowed studying the details of how mechanical mixing of metals occurs on the micro-scale level. The result is the discovery that some basic features in shear deformation behavior of the metallic systems at this level are similar to those of geological phenomena such as rock folding, boudinage, or the development of SC fabrics in layered and sheared rocks^28, 29^. Rock folding occurs at the macro-scale regime on kilometer length scales and over millions of years, whereas the deformation of the metallic systems occurs on micrometers and for minutes. The presented computer simulations thus offer a powerful tool for understanding the origin of morphological evolution and deformation behavior of ductile multiphase metallic systems under shear, both in crystalline and amorphous systems, opening new opportunities to study various materials processing techniques as well as tribological events such as frictional wear.

## Methods

### High-pressure torsion deformation

High-purity foils of Cu (99.8%), Ag (99.95%) and Al (99.45%) all purchased from Alfa Aesar, with each foil having a thickness of 25 µm, were used as the starting materials. For the experiments, stacks of foils consisting of alternating 11 Cu and 10 Al or Ag foils, with dimensions of 20 × 20 mm, were used. The entire stacks had a total initial thickness of 525 µm.

The HPT deformation process on each stack was performed using a pair of anvils made of Böhler S390 Microclean, each having 200-µm-deep circular cavities with a diameter of 10 mm. During HPT process, the stack is initially pressed between the anvils and while holding the pressure at 4.5 GPa, the lower anvil rotates against the upper one at a velocity of 1 r.p.m. and thereby applying a horizontal shear stress to the sample. To put the pressure that is applied in this stage of the experiment into perspective, it is roughly equivalent to the pressure that would be felt at a depth of 150 km below the surface of the Earth^30^. The HPT processing was performed for five revolutions in case of Ag/Cu and for three revolutions in case of Al/Cu. The applied rotational velocity corresponds to a maximum strain rate of 1.3 s^−1^. The processed disks had a final thickness of 400 µm. Using equation (1) and the values of *r* = 5 mm, *t* = 400 µm, and corresponding *N* (5 for Ag/Cu and of 3 for Al/Cu), the accumulated strain can be calculated; using these values, a strain of 393 for Ag/Cu and 236 for Al/Cu were computed.

### 3D X-ray synchrotron tomography

The 3D X-ray synchrotron tomography images were acquired at the beamline ID19 of the European Synchrotron Radiation Facility (ESRF, Grenoble, France)^31^. For this purpose, a parallelepiped-shaped volume section was used, which was cut along the radius of the processed disk by erosion cutting and the cut surfaces were then subsequently wet-polished with a fine abrasive paper.

For the Ag/Cu specimen, the wiggler source of the beamline was chosen (gap: 40 mm) in order to access photon energies that would be sufficient to transmit through the dense sample. A combination of a diamond absorber with 5.6 mm Al and 0.7 mm Cu in the beam path suppressed the lower part of the emitted spectrum, while 60 compound-refractive lenses downstream of the filters collimated, having the desired photon energy on the detector with nominal value of 80 keV (quasi-inline monochromator configuration). As detector, a radiation hard system (OptiquePeter, Lentily, France) was chosen where a ×10/0.28NA Mitutoyo long-working distance objective projects the luminescence image of a 25 µm GGG:Eu single-crystal scintillator (Eu-doped Gd_3_Ga_5_O_12_) via an ×3.3 eye piece onto the chip of a CCD camera (ESRF-in-house type FReLoN e2v with a corresponding 230–42 chip)^32^. The effective pixel size of the detector is 0.52 µm while the spatial resolution is in the order of 2 µm. The sample-detector distance was 160 mm. A total of 2000 projection images were acquired over 360 degrees to ensure a good tomographic reconstruction as performed via the ESRF-inhouse software PyHST_2 via the common filtered back-projection algorithm^33^. In order to further reduce noise and improve the contrast, a single-distance phase retrieval was applied following Paganin’s approach^34^.

For the Al/Cu specimen, the U17.6 undulator insertion device (gap: 11.5 mm) was combined with a 1-mm diamond absorber, 2.8 mm Al, 0.4 mm Cu and 12 compound-refractive lenses for a nominal photon energy of 53 keV. The detector was a classical visible-light microscopy (×10/0.3NA Olympus objective with ×2 eye piece), which projects the luminescence image of a 10-µm GGG:Eu single-crystal scintillator onto the chip of a FReLoN CCD camera (type A7899). The effective pixel size is 0.64 µm (2 µm spatial resolution). The other experimental parameters and the data processing chain remained the same as for the Ag/Cu specimen.

### Finite element simulation

The simulations described in this paper are computed with the thermo-mechanical marker-and-cell finite element code MVEP2^35, 36^ that simulates large strain deformation of creeping high viscous fluids.

In the problem we consider, shear deformations are significantly larger than volumetric deformation, which are described by the conservation of mass and momentum for slowly deforming fluids:

3 $$(\partial v_i)/(\partial x_i) = 0,$$

4 $$(\partial \sigma _{ij})/(\partial x_j) = \rho g_i,$$

where *v*_*i*_ denotes velocity, *σ*_*ij*_ = −*Pδ*_*ij*_ + *τ*_*ij*_ the components of the stress tensor with *δ*_*ij*_ being the Kronecker delta, *P* pressure, *τ*_*ij*_ the components of the deviatoric stress tensor, *g*_*i*_ gravitational acceleration, *ρ* density, and the Einstein notation is used.

We employ a viscous rheology configuration given by:

5 $${{\dot \gamma }}_{ij} = \frac{{{\it{\tau }}_{ij}}}{{2{\it{\eta }}_{{\mathrm{eff}}}}},$$

where ${{\dot \gamma }}_{ij}$ are the components of the strain rate tensor^37^, and *η*_eff_ the effective viscosity.

The effective viscosity is computed according to the relation,

6 $${\it{\eta }}_{{\mathrm{eff}}} = {\it{\eta }}_{\mathrm{0}}\left( {\frac{{{{\dot \gamma }}_{{\mathrm{II}}}}}{{{{\dot \gamma }}_0}}} \right)^{\frac{1}{n} - 1},$$

where *n* is a power-law exponent (a material constant), ${{\dot \gamma }}_{{\mathrm{II}}} = (0.5{{\dot \gamma }}_{ij}{{\dot \gamma }}_{ij})^{0.5}$, the second invariant of the strain rate tensor and *η*_0_ the reference viscosity, and ${{\dot \gamma }}_{\mathrm{0}}$ a reference strain rate (taken as 1 s^−1^ here). In this paper, in all simulations, a viscosity cutoff of 10 Pa·s and 10^7^ Pa·s is employed. The code uses a marker-and-cell approach to track material properties and large strains, uses direct solvers to solve the weak form of the governing equations and has been extensively tested for folding instabilities.

To determine the effect of absolute value of characteristic viscosity *η*_0_ on the morphological evolution, a few simulations were run for two different cases: *η*_0_ = 10^5^ and *η*_0_ = 10^7^ Pa·s. It is found that the absolute value of the characteristic viscosity has no influence on the dynamics of evolution and the key determining parameter is the viscosity contrast VC between the phases.

### Data availability

The data that support the findings of this study are available from the corresponding authors upon reasonable request.

## Electronic supplementary material

Supplementary Information

Description of Additional Supplementary Files

Supplementary Movie 1

Supplementary Movie 2

Supplementary Movie 3

Supplementary Movie 4

Supplementary Movie 5

Supplementary Movie 6
