# Supplementary material for: Mixing instabilities during shearing of metals
Source: Nat Commun. 2017 Nov 20;8:1611. doi: 10.1038/s41467-017-01879-5 (PMC5694766; doi:10.1038/s41467-017-01879-5)
Supplement: Supplementary file 1 — Supplementary Information [file 41467_2017_1879_MOESM1_ESM.pdf]

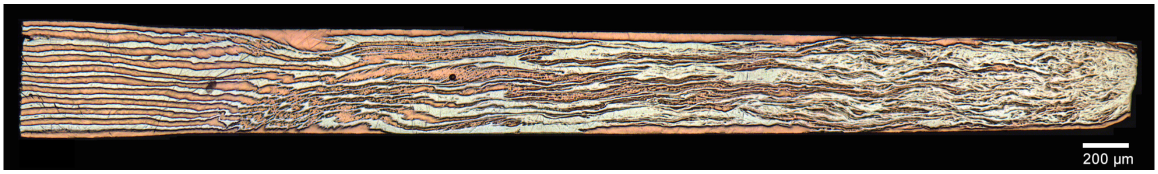

Supplementary Fig. 1 Optical microscopy on HPT-deformed Ag/Cu multilayer after 5 revolutions at 1 rpm. The cross-section is made along the radius of the deformed disk. The left side was at the center and the strain increases towards the right. Note that here the sense of shear is perpendicular to the page, while Fig. 2 and Supplementary Movies 1 and 2 depict cross-sections parallel to the shear direction.

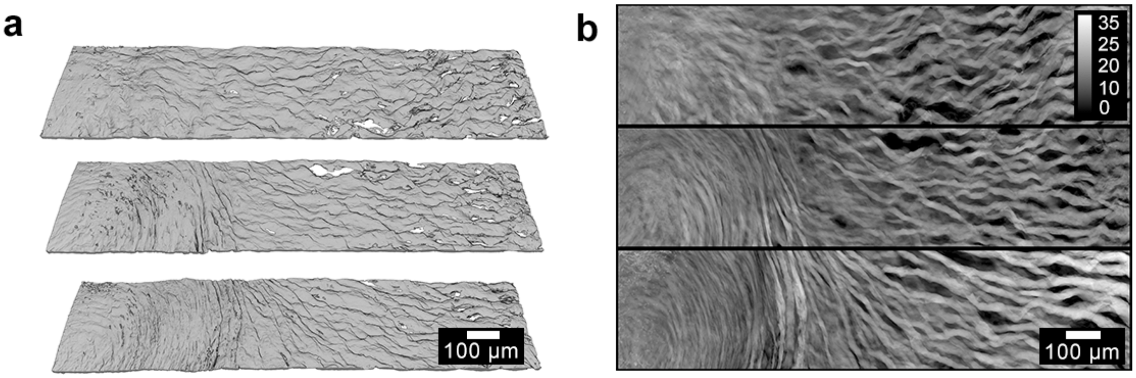

Supplementary Fig. 2 HPT-deformed Al/Cu multilayer after 3 revolutions at 1 rpm acquired by 3D X-ray synchrotron tomography. **a**, Perspective projection of three selected successive Cu-layers within the multilayer, which is shown in Fig. 2f–j. The left side was at the center of the disk and the strain increases towards the right. In all three layers, the layers are depicted before they lose their continuity, due to substantial shear mixing occurring towards edge of the disk. The Al interlayers are rendered transparent for better visibility. **b**, Top view of the Cu-layers shown in (a). The gray scale represents the height profile in µm. This figure shows that, when viewed from the top, vortices appear as bands. The average size of the band-like structures (vortices in the frontal view) gets larger as the strain increases from the left to the right of the image. This is consistent with the fact that the vortices become larger as the strain increases.

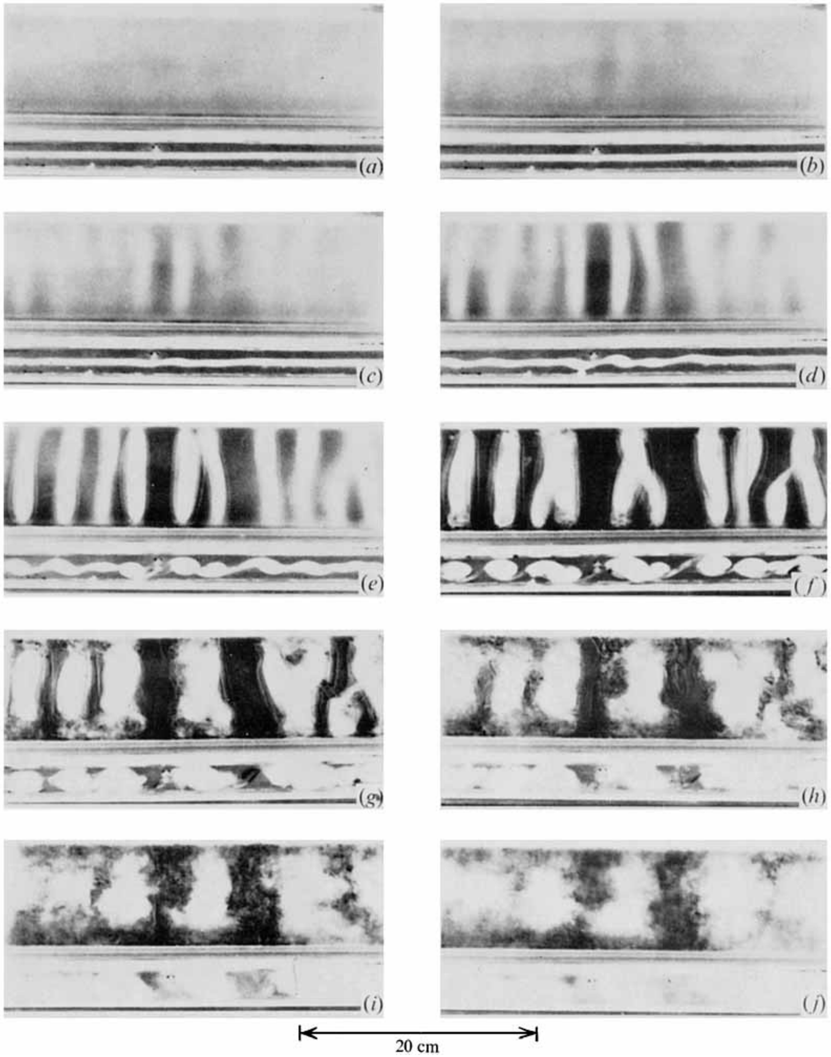

Supplementary Fig. 3 Development of Kelvin-Helmholtz instability at the interface between water and brine with a layer of dye at the interface[1]. The photographs are in negative so that the dye appears white. The upper part of each frame shows the top view and the lower part is frontal view. The first photograph is taken at 2.50 s after shearing, and the successive photographs are taken at 0.20 s intervals. Note the similarities of microstructural features in this figure with those of Al/Cu multilayer [Fig. 2f–j (frontal view), and Supplementary Fig. 2b (top view)].

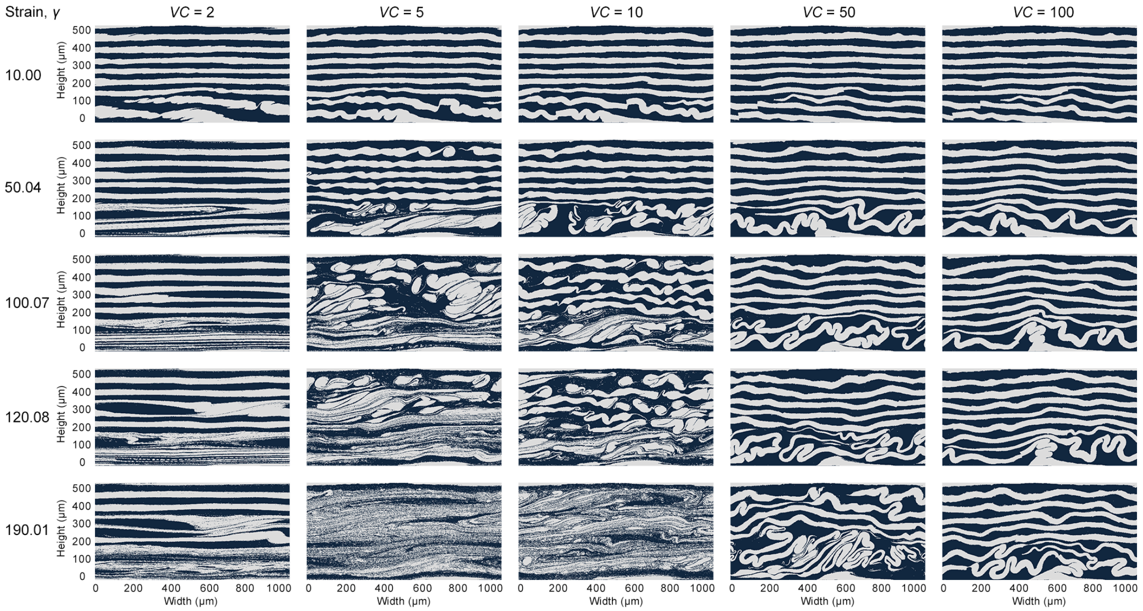

Supplementary Fig. 4 Dependence of morphological evolution on the viscosity contrast VC. The stress exponent is set to be constant at a value of n=3. Note that the lower the VC, the smaller the wavelength of the folds and vice versa. Also, note that no vortex-like structures develop in case of VC=2.

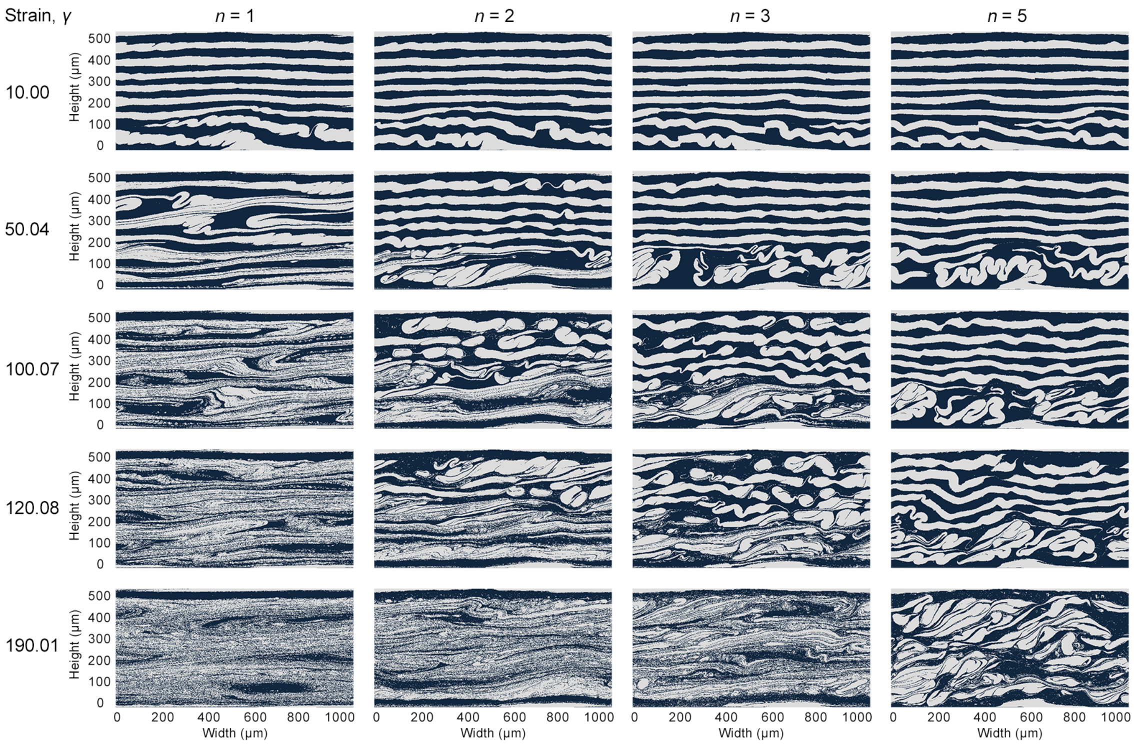

Supplementary Fig. 5 Dependence of morphological evolution on the stress exponent *n*. The viscosity contrast is set to be constant at a value of VC=10. Note that the lower the *n*, the smaller the wavelength of the folds and vice versa.

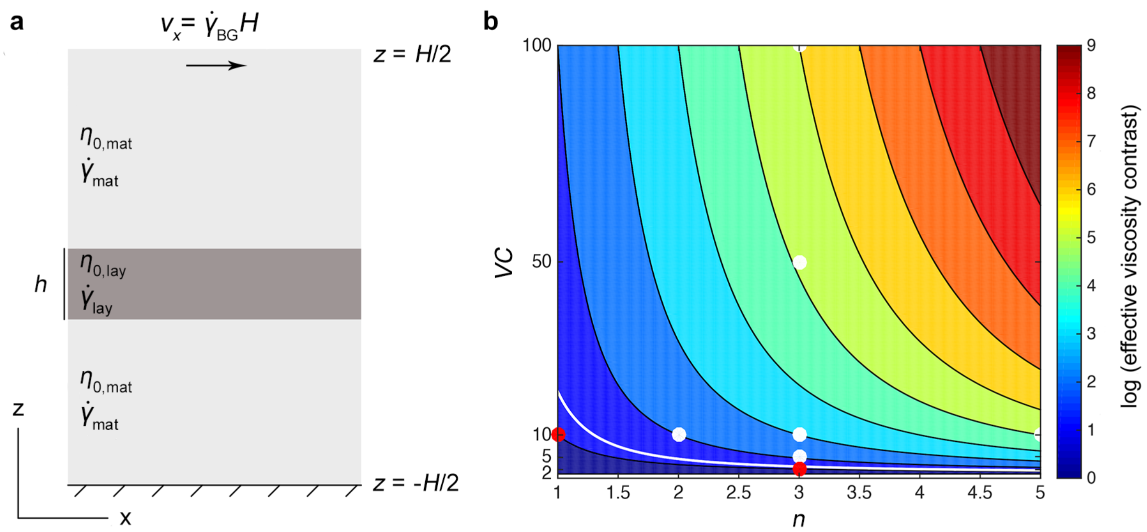

Supplementary Fig. 6 Effective viscosity contrast as a function of VC and *n*. **a**, 1D simple shear setup in which a highly viscous nonlinear material is embedded in a lower viscous matrix. The whole system is subjected to simple shear with a background strain rate of $\dot{\gamma}_{BG}$. **b**, Contours indicate the logarithm of the computed effective viscosity contrast between the layer and the matrix for the 1D simple shear setup, with the white line indicating an effective viscosity contrast of 20. Circles correspond to the results of numerical simulations for a multilayer stack (Supplementary Figs. 4 and 5), which were visually inspected to determine whether vortex-like structures are formed (white circles) or not (red circles).

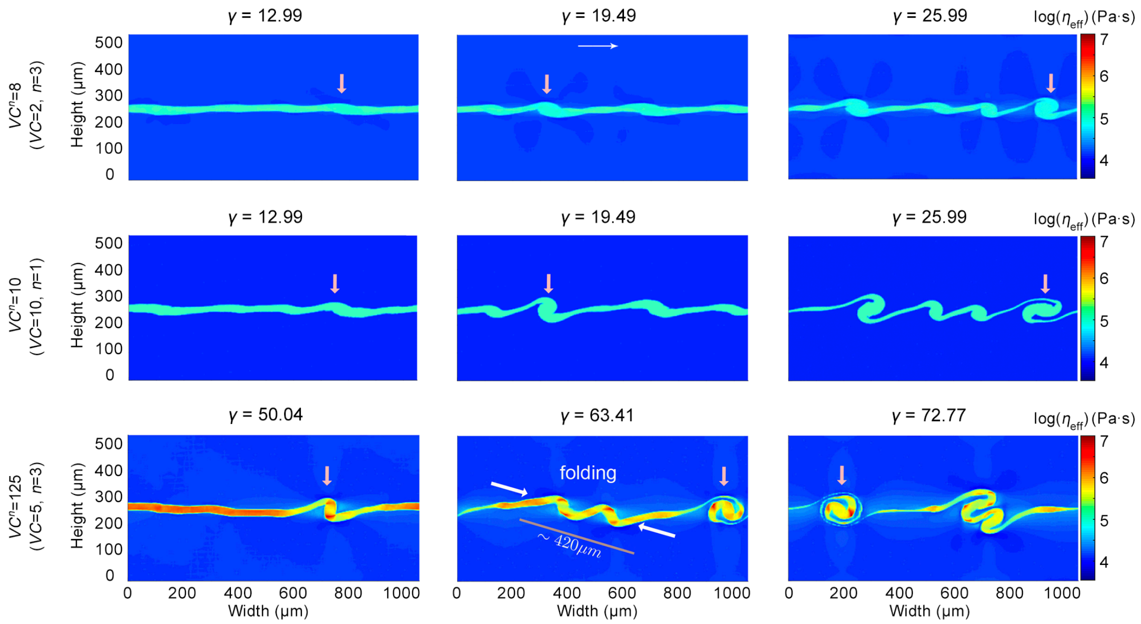

Supplementary Fig. 7 Evolution of effective viscosity in a single layer under shear for different material parameters. The parameters are arranged such that the effective viscosity contrast increases from top to bottom. Colors indicate the effective viscosity. The initial layer was identical to the one in Supplementary Fig. 8b. Whereas for smaller viscosity contrasts, vortices form in a purely kinematic/geometric manner, an intermediate folding instability occurs for larger effective viscosity contrasts which forms a vortex-like structure at a later stage. Also note that the amount of strain required to form the first structure increases with increasing viscosity.

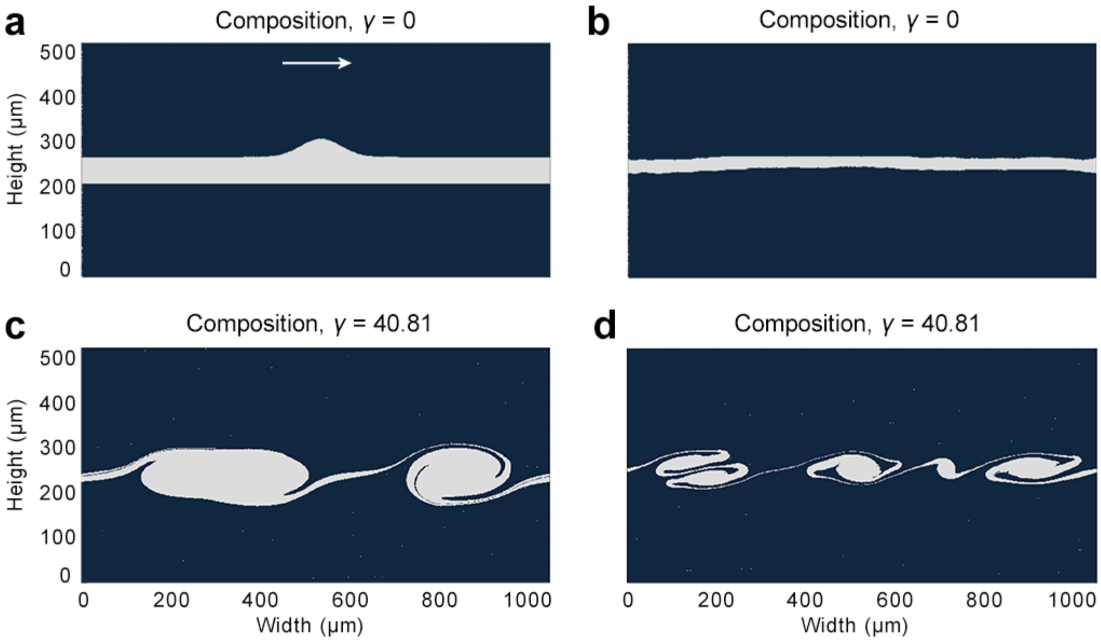

Supplementary Fig. 8 Morphological evolution of two single layers with different initial geometries. The simulations are for (VC=2, *n*=3). The top and bottom rows show the initial morphology and morphology after a strain of 40.81, respectively. The thicker layer (left) results in larger vortex-like structures.

Supplementary Note 1: Feasibility of Kelvin-Helmholtz instability in solids

Our aim here is to evaluate the feasibility of Kelvin-Helmholtz (KH) instability in solids under our experimental conditions. Here we do the estimations for Cu, which is the phase with apparent KH instability features in Al/Cu system, but the calculation is similar for other phases. Since KH instability is caused by inertial forces, it requires the fluid to be turbulent. A flow becomes turbulent as its corresponding Reynolds number exceeds a critical value. The Reynolds number Re is defined as

$$Re = \frac{\rho v L}{\eta_{\mathrm{eff}}} \quad (1)$$

with ρ being the density (kg m^{-3}), v the characteristic flow velocity (m s^{-1}), L a characteristic linear dimension (m), and η_{eff} the effective viscosity of the fluid (Pa·s). The critical Reynolds number of a fluid flowing through a circular pipe is Re =2300, above which flow is turbulent and below which it is laminar[2].

Considering the radius of the disk in our experiments (5 mm), as an upper bound for L, and substituting it in Supplementary Equation 1, together with Re =2300, ρ(Cu) =8920 kg m^{-3}, and assuming an effective viscosity as low as η_{eff} =10^{4} Pa·s, implies that the material should flow at a velocity of v >500 km s^{-1}, to be in the turbulent regime. For comparison, the maximum shear velocity in our experimental setup is achieved at r = 5 mm, which for the rotational velocity of 1 rpm used in our experiments is equal to $2\pi r / 60 \approx 500$ μm s^{-1}. Hence, KH instability cannot be accounted as a viable mechanism for producing the vortices observed in Al/Cu multilayer.

Supplementary Note 2: Effective viscosity contrast and vortex formation

The numerical simulations suggest that the effective viscosity contrast between the weak and strong layers has an influence on whether vortices form or not. In a power-law viscous material, the effective viscosity depends on the local strain rate. Therefore, it cannot be directly estimated from the background strain rate. Instead, it is useful to consider a 1D-setup under simple shear with a single high viscosity layer of thickness h that is embedded in a lower viscous matrix in a system with overall thickness H (Supplementary Fig. 6a).

In 1D, the governing equations are:

$$\frac{\partial \sigma_{xz}}{\partial z} = 0 \tag{2}$$

$$\dot{\gamma}_{xz} = \frac{1}{2}\frac{\partial v_x}{\partial z} \tag{3}$$

$$\sigma_{xz} = 2\eta_{\mathrm{eff}}\dot{\gamma}_{xz} \tag{4}$$

$$\eta_{\mathrm{eff}} = \eta_0 \left|\frac{\dot{\gamma}_{xz}}{\dot{\gamma}_0}\right|^{\frac{1}{n}-1} \tag{5}$$

whereas the boundary conditions are

$$v_x(z = -\frac{H}{2}) = 0\,,\ v_x(z = \frac{H}{2}) = \dot{\gamma}_{\mathrm{BG}} H \tag{6}$$

where $\dot{\gamma}_{\mathrm{BG}}$ is the applied background strain rate. From force balance it follows:

$$\sigma_{xz} = \mathrm{constant} \tag{7}$$

If $\dot{\gamma}_{\mathrm{mat}}$ is the strain rate in the matrix and $\dot{\gamma}_{\mathrm{lay}}$ the strain rate in the layer, and $\eta_{0,\mathrm{mat}}$, $\eta_{0,\mathrm{lay}} = \mathrm{VC}\eta_{0,\mathrm{mat}}$ the corresponding viscosity prefactors in the matrix and layer, respectively, it follows from force balance that

$$\eta_{0,\mathrm{mat}} \left|\frac{\dot{\gamma}_{\mathrm{mat}}}{\dot{\gamma}_0}\right|^{\frac{1}{n}} = \eta_{0,\mathrm{lay}} \left|\frac{\dot{\gamma}_{\mathrm{lay}}}{\dot{\gamma}_0}\right|^{\frac{1}{n}} \tag{8}$$

or

$$\dot{\gamma}_{\mathrm{mat}} = \left(\frac{\eta_{0,\mathrm{lay}}}{\eta_{0,\mathrm{mat}}}\right)^n \dot{\gamma}_{\mathrm{lay}} = \mathrm{VC}^n \dot{\gamma}_{\mathrm{lay}} \tag{9}$$

The effective viscosity contrast between the layer and the matrix is

$$\frac{\eta_{\mathrm{eff,lay}}}{\eta_{\mathrm{eff,mat}}} = \frac{2\eta_{0,\mathrm{lay}}\left|\dot{\gamma}_{\mathrm{lay}}\right|^{\frac{1}{n}-1}}{2\eta_{0,\mathrm{mat}}\left|\dot{\gamma}_{\mathrm{mat}}\right|^{\frac{1}{n}-1}} = \frac{\eta_{0,\mathrm{lay}}\left|\dot{\gamma}_{\mathrm{lay}}\right|^{\frac{1}{n}-1}}{\eta_{0,\mathrm{mat}}\left|\mathrm{VC}^n\dot{\gamma}_{\mathrm{lay}}\right|^{\frac{1}{n}-1}} = \frac{\eta_{0,\mathrm{mat}}\mathrm{VC}\left|\dot{\gamma}_{\mathrm{lay}}\right|^{\frac{1}{n}-1}}{\eta_{0,\mathrm{mat}}\mathrm{VC}^{1-n}\left|\dot{\gamma}_{\mathrm{lay}}\right|^{\frac{1}{n}-1}} = \mathrm{VC}^n \tag{10}$$

A comparison of the predicted viscosity contrast with the results of the numerical simulations in a multilayer stack shown in Supplementary Figs. 4 and 5 indicates that

vortex-like structures form once the viscosity contrast is larger than ~20-50 (Supplementary Fig. 6). However, simulations with smaller viscosity contrasts result in faster mixing without clear intermediate vortex-like structures. This comparison also shows that larger amount of strains are required for mixing to occur for larger effective viscosity contrasts.

Simulations with a single layer show that for lower viscosity contrasts, vortex-like structures still form but in a kinematic manner, as a result of the applied simple shear (Supplementary Fig. 7). For a larger effective viscosity contrast, a folding instability occurs before developing to a larger vortex-like structure (Supplementary Fig. 7).

Folding of a single layer with a power-law material embedded in a power-law matrix under pure shear is known to have the following dominant wavelength expression[3]:

$$\lambda_{\mathrm{dom}} = 2\pi H_{\mathrm{layer}} \left(\frac{\mathrm{VC}}{6n^{1/2}} \right)^{\frac{1}{3}} \tag{11}$$

where H_{layer} is the thickness of the layer, which is ~40 μm for the simulations shown in Supplementary Fig. 7. For the case that shows intermittent folding in this figure (VC =5, n =3), we obtain λ_{dom} =197 μm, which is in good agreement with the observed fold wavelength.

Supplementary References

1. Thorpe, S. A. Experiments on the instability of stratified shear flows: miscible fluids. *J. Fluid Mech.* **46,** 299–319 (1971).

2. Çengel, Y. A. & Cimbala, J. M. *Fluid Mechanics: Fundamentals and Applications*. (McGraw Hill Higher Education, 2006).

3. Fletcher, R. C. Wavelength selection in the folding of a single layer with power-law rheology. *Am. J. Sci.* **274,** 1029–1043 (1974).
