# Supplementary material for: Mixing instabilities during shearing of metals
Source: Nat Commun. 2017 Nov 20;8:1611. doi: 10.1038/s41467-017-01879-5 (PMC5694766; doi:10.1038/s41467-017-01879-5)
Supplement: Supplementary file 2 — Description of Additional Supplementary Files [file 41467_2017_1879_MOESM2_ESM.docx]

**Description of Additional Supplementary Files**

File Name: Supplementary Movie 1

Description: Morphological evolution during shearing in Ag/Cu. The arrow at the top shows the direction of the shear. Some selected snapshots are shown in Fig. 2a–e. The principle used to visualize the evolution is alike the one used in flipbooks: a series of slightly shifted slides played in sequence.

File Name: Supplementary Movie 2

Description: Morphological evolution during shearing in Al/Cu. The arrow at the top shows the direction of the shear. Some selected snapshots are shown in Fig. 2f–j. The same principle as Movie S1 is used here.

File Name: Supplementary Movie 3

Description: Finite element simulation of morphological evolution for (*VC* =10, *n* =3). The arrow at the top shows the direction of the shear. Some selected snapshots are shown in Fig. 3.

File Name: Supplementary Movie 4

Description: Finite element simulation of evolution of strain rate for (*VC* =10, *n* =3). The arrow at the top shows the direction of the shear.

File Name: Supplementary Movie 5

Description: Finite element simulation of evolution of effective viscosity for (*VC* =10, *n* = 3 ). The arrow at the top shows the direction of the shear.

File Name: Supplementary Movie 6

Description: Evolution of morphology (left) and strain rate (right) for a perturbation in case of (*VC* =5, *n* =3). The arrow at the top shows the direction of the shear. Some selected snapshots are shown in Fig. 4.
